# Evaluation of Culturally-Familiar Odorants for a Persian Smell Identification Test

**Published:** 2018-01

**Authors:** Seyed Kamran Kamrava, Maryam Jalessi, Shaghayegh Ebrahimnejad, Sahand Ghalehbaghi, Elahe Amini, Alimohamad Asghari, Farhad Rafiei, Mohammad Farhadi

**Affiliations:** 1 *ENT and Head and Neck Research Center and Department, Hazrat Raoul Akram Hospital, Iran University of Medical Sciences, Tehran, Iran.*; 2 *Skull Base Research Center, ENT and Head and Neck Research Center, Hazrat Rasoul Akram Hospital, Iran University of Medical Sciences, Tehran, Iran.*

**Keywords:** Culture, Identification, Smell, Odorants

## Abstract

**Introduction:**

Processing odor information by the olfactory system depends greatly on the odor concentration. In order to use an odorant in a smell identification test (SIT), the minimum identification concentration (MIC) needs to be determined.

**Materials and Methods:**

This study was conducted in 60 healthy native individuals aged 20 to 60 years, selected from patients’ companions in a tertiary hospital. In the first step, 25 odorants were presented to evaluate familiarity among the subjects. Then, the MICs for the eligible odorants were measured using the ascending method of limits.

**Results:**

Out of 25 odorants, only one (cacao) was distinguished by less than 70% of the subjects, and was therefore removed from the list. The MICs of the remaining 24 odorants ranged from 6.87±2.74% for menthol to 27.62±18.98% for cantaloupe. There was significant correlation between age and the MIC only for coffee (P=0.02, r=−0.300). There was a significant difference in MIC between men and women only for hazelnut (P=0.03).

**Conclusion:**

We present the MICs of 24 culturally-familiar odorants in a sample of the Persian population in a SIT.

## Introduction

The sense of smell strongly affects human quality of life and health (1). Evaluation of a patient’s olfactory function is an important step in diagnosing and treating the olfactory dysfunctions, and also in the early detection of some neurological diseases (2,3). Newly developed olfactory tests are mainly subjective psychophysical tests that rely on subject detection, identification or discrimination. These tests are easy to use and are more cost-beneficial compared with electrophysiological tests (4).

Smell identification tests (SIT) were the first olfactory tests to gain popularity. Among them, the University of Pennsylvania Smell Identification Test (UPSIT)and its modifications are most commonly, and have been evaluated for applicability in several countries. Another widely available SIT is the Sniffin' Sticks test, which consists of felt-tip pens filled with odorants. Removal of the cap will release the odor. This test originally developed and validated in Germany, and is now also validated in several countries (5–10).

For an odorant to be used in a SIT, the prior condition is to be recognizable by more than 70–75% of the subjects tested (11,12). Many investigations have shown that the identification of an odorant closely depends on social and cultural factors (13,14). In a study by Kamrava et al. (15), more than 50% of the odorants used in the UPSIT were not familiar to the Iranian population, and thus needed to be replaced by more familiar ones.

Furthermore, processing odor information by the olfactory system depends largely on the odor concentration (16). The ability to recall an odorant or define the intensity is directly linked to the minimum identification concentration (MIC) of that odorant in each population (17). MIC is a characteristic of a chemical agent, and the response would be consistent at all higher concentrations (18).However, establishing a MIC for an odorant is not a straight-forward task, and no clear consensus is available on how to quantify an odorant )^19^,^20^.( Different populations may have particular cultural odorant materials, and the recognizable concentration of these materials may differ between communities.

In the present study, we attempted to find culturally-familiar odorants and the MICs of each one in order to be able to use them in a new SIT.

## Materials and Methods

This study was conducted in a group of healthy Iranian volunteers, aged 20 to 60 years, selected from patients’ companions in a tertiary hospital. The subjects had no complaint of nasal obstruction, recent upper respiratory tract infection or allergy attack, nasal and sinus surgeries, head trauma or any systemic chronic diseases such as liver or renal dysfunction or history of neurological or psychological problems (except for mild depression or anxiety). Any history of use of medications affecting olfaction was considered an exclusion criterion. This study was performed in accordance with the Declaration of Helsinki, and approved by the ethics committee of the Ear, Nose and Throat (ENT) and Head and Neck Research Center. Informed consent was obtained from all participants.

The principles for selection of the odorants were familiarity with all odorants-describing items, similarity in intensity and hedonic tone, and corrected identification rate more than 70% in healthy subjects (5,11,12). Odor familiarity rate was evaluated through a list of multiple choices. In order to select the appropriate odorants, nine odorants that were shown to have more than 70% identification rate in a previous study on Iranian population were adopted (15). Six odorants that are known to be stimulators of trigeminal nerve were also added to the list.

Then, 60 healthy subjects were asked to rate 40 odorants using a questionnaire adopting a Likert-type scale ranging from 0 to 5 (0=unfamiliar, 5=very familiar). The results of each odorant were converted to a percentage, and odorants with a familiarity rate of greater than 70%, were selected.

In the second phase, 25 eligible odorants (by Magnolia Co., Iran) were presented to 10 subjects to confirm their familiarity in a pilot study. Then, all subjects were presented with the odorants. The subjects were asked to not eat, drink or smoke 15 min prior to the test. The odorants were presented in uniform pens with tampons filled with odorants that had been labeled with different codes at the bottom. The cap was then removed for 3 s, and the patient was asked to sniff. Each marker was placed 1 to 2 cm away from the nostrils. The subjects were asked to choose the correct answer from the list of multiple choices. The time interval between presenting the various odorants was 30 s. Those odorants that were identified correctly in more than 70% of the subjects were selected for the second phase of the study. In the third phase, the odorants were presented to the subjects in five different concentrations (6.25%, 12.5%, 25%, 50%, and 75%) with the same disciplines adopted in the first phase. The concentrations were presented to the subjects from the lowest to the highest for each odorant (ascending method of limits). The subjects were asked to identify the odorant in a questionnaire. If the answer was incorrect, a higher concentration was presented until the correct answer was reached. This concentration was assumed to be the MIC for that odorant, and was measured in each person for all of the odorants. All procedures were followed by one expert in a specified quiet room (smell laboratory) using standard methods (21–24).

The results were presented as mean ± standard deviation (SD) for quantitative variables, and frequency (percentage) for categorical variables. Continuous variables were compared using t-test, analysis of variance (ANOVA) test or non-parametric Mann-Whitney U test or Kruskal-Wallis test whenever the data did not appear to have normal distribution. Categorical variables were compared using the chi-square test. For the statistical analysis, the statistical software SPSS version 22.0 for windows (SPSS Inc., Chicago, IL) was used. P-values of 0.05 or less were considered statistically significant.

## Results

Sixty healthy individuals (28 men and 32 women) with a mean age of 37.5±9.7 years, ranging from 20 to 58 years were included (men: 38.61±9.86 years [23 to 57 years], women: 36.53±9.60 years [20 to 58 years]). A final list of odorants consisting of 25 descriptors and 31 distractors was developed. Out of 25 odorants that were evaluated, one odorant (cacao) did not reach the correct identification rate of 70% (61.6%), and was thus removed from the list. The percentages and numbers of corrected detections of each odorant are presented in Table 1. 

**Table 1:** Numbers and percent of corrected detection of the 25 odorants

**Odorant**	**Number and percent**	**Odorant**	**Number and percent**
Coffee	60 (100%)	Orange	60 (100 %)
Vinegar	59 (98.3%)	Saffron	59 (98.3%)
Banana	60 (100%)	Cantaloupe	58 (96.6%)
Mint	55 (91.6%)	Smoke	59 (98.3%)
Coconut	59 (98.3%)	Rosewater	60 (100%)
Cucumbers	53 (88.3%)	Cardamom	56 (93.3%)
Onion	53 (88.3%)	Honey	44 (73.3%)
Cinnamon	57 (95 %)	Crud	53 (88.3%)
Cacao	37 (61.6%)	Hazelnut	51 (85%)
Apple	56 (93.3%)	Garlic	59 (98.3%)
Menthol	58 (96.6%)	Butter	60 (100%)
Pineapple	60 (100%)	Lemon	60 (100%)
Vanilla	53 (88.3%)		

The MIC for the remaining 24 odorants evaluated ranged from 6.87±2.74% for menthol to 27.62± 18.98% for cantaloupe. The MIC of the odorants are shown in Table 2. Out of all odorants, only the MIC of hazelnut was significantly different in men and women (6.25±0.01 vs. 7.81±3.88,respectively, P=0.03).

Comparing the MICs of the odorants across age groups, a significant difference was found for lemon (P=0.01). The mean MIC for each odorant in each age group is shown in Table 3. There were significant correlations between age and mean MIC for coffee (P=0.02, r =−0.30) and lemon (P=0.05, r =−0.26). These MICs were then rounded up to the next higher concentration. The percentage of correct identification at these concentrations was 71–98.3%, and thus considered as the MIC for each odorant.

**Table 2:** Mean±standard deviation (SD) of minimum identification concentration of each odorants

**Odorant**	**Total Mean±SD**	**Men Mean±SD**	**Women Mean±SD**
Coffee	17.60±12.15	19.64±13.03	15.82±11.22
Vinegar	10.52±6.35	10.49±5.90	10.54±6.81
Banana	7.60±6.20	8.03±8.30	7.22±3.58
Mint	7.81±3.92	8.48±5.16	7.22±2.30
Coconut	12.50±9.34	12.94±6.34	12.10±11.44
Garlic	8.64±3.65	8.48±3.04	8.78±4.15
Curd	19.80±15.57	20.98±15.61	18.75±15.72
Apple	10.52±9.16	9.82±6.68	11.13±10.96
Cinnamon	11.86±10.42	12.26±11.69	11.52±9.40
Menthol	6.87±2.74	7.14±3.69	6.64±1.53
Cucumbers	7.18±3.00	6.91±1.96	7.42±3.70
Pineapple	12.91±9.61	12.05±9.46	13.67±9.84
Lemon	22.52±17.90	23.43±17.31	21.66±18.69
Orange	7.39±3.72	7.81±4.03	7.03±3.45
Saffron	9.06±7.03	8.70±4.28	9.37±8.83
Smoke	10.52±6.03	12.05±6.78	9.17±5.01
Rosewater	23.95±19.90	24.55±17.83	23.43±21.82
Cardamom	7.29±3.66	6.69±1.63	7.81±4.76
Honey	14.11±13.32	12.96±9.79	15.12±15.87
Vanilla	14.16±11.72	15.62±11.96	12.89±11.55
Hazelnut	7.08±2.92	6.25±.01	7.81±3.88
Cantaloupe	27.62±18.98	27.60±20.76	27.64±17.60
Butter	7.70±3.87	8.03±4.11	7.42±3.70
Onion	8.16±7.23	8.37±6.83	7.97±3.28

**Table 3:** Mean±standard deviation (SD) of each odorant’s minimum identification concentration in age groups

**Odorants**	**20–30 years** **Mean±SD**	**30–40 years** **Mean±SD**	**40–50 years** **Mean±SD**	**50–60 years** **Mean±SD**	**P-value**
Coffee	23.82±15.00	16.87±13.15	14.14±7.16	13.75±6.84	0.09
Vinegar	12.10±7.02	10.62±6.75	9.86±6.00	7.50±2.79	0.51
Banana	7.42±2.51	8.43±9.78	7.23±4.30	6.25±.00	0.88
Mint	7.42±4.68	6.87±1.92	9.21±4.82	7.50±2.79	0.29
Coconut	12.89±11.74	10.93±6.68	14.80±10.46	8.75±3.42	0.47
Garlic	7.81±2.79	8.12±2.93	9.53±4.82	10.00±3.42	0.39
Curd	26.95±19.19	15.31±11.55	20.06±15.53	12.50±8.83	0.11
Apple	9.37±6.45	10.62±10.35	12.17±10.91	7.50±2.79	0.71
Cinnamon	15.62±14.79	11.84±11.00	9.86±4.80	7.50±2.79	0.30
Menthol	7.81±4.84	6.56±1.39	6.57±1.43	6.25±.00	0.46
Cucumbers	7.81±2.79	6.25±.00	7.56±4.45	7.50±2.79	0.40
Pineapple	12.10±8.05	13.12±10.31	14.14±11.38	10.00±3.42	0.83
Lemon	33.98±19.49	16.77±14.13	17.70±11.98	25.00±28.98	0.01
Orange	6.64±1.56	7.81±4.47	6.57±1.43	11.25±8.14	0.06
Saffron	10.93±11.52	9.06±5.90	7.56±2.61	8.75±3.42	0.58
Smoke	9.37±5.10	10.62±5.77	10.85±6.85	12.50±7.65	0.76
Rosewater	29.68±26.26	19.06±15.23	25.32±18.45	20.00±18.43	0.42
Cardamom	6.64±1.56	6.56±1.39	7.89±4.58	10.00±8.38	0.21
Honey	14.58±17.93	14.80±11.06	13.48±13.21	12.50±7.65	0.98
Vanilla	10.15±6.40	15.62±13.37	17.76±13.54	7.50±2.79	0.13
Hazelnut	6.64±1.56	7.18±2.28	7.56±4.45	6.25±.00	0.73
Cantaloupe	32.14±19.28	26.64±19.96	26.64±19.96	31.25±21.65	0.64
Butter	6.64±1.56	8.12±4.57	8.22±4.68	7.50±2.79	0.62
Onion	10.63±9.51	8.93±4.99	8.53±3.60	8.32±4.01	0.47

## Discussion

SITs are currently the most popular olfactory tests that are available in various versions in different countries (25–27). In the United States, UPSIT is the SIT that is most commonly used in studies focusing on olfaction. This test is shown to be able to clearly differentiate between persons with normal olfactory ability and those who have well-documented olfactory dysfunction (14).

However, there are some limitations regarding the use of UPSIT in other countries. When the standard UPSIT was used in Japanese normal subjects, the identification rates of some odorants were quite low. For this reason, a cross-culturally modified UPSIT was developed in the Japanese population (28). This also led to a British version and an international version of the UPSIT (27,29). A study in Iran using UPSIT demonstrated most of the odorants of this test were not familiar in this population, with more than 50% of odorants of this test having less than 70% correct identification rate (15). Therefore, it was necessary to develop and adopt a SIT that is adapted to the Iranian culture.

The next step in adopting an odorant in a SIT is to determine the MICs of the odorants that are going to be used. Through standardization of the method of sample presentation and minimizing of the extraneous sensory interference, we tried to achieve a higher accuracy in determining the MIC. Because determining the optimal MICs may directly depend on the sensitivity of human olfaction, we tried to select subjects with the healthiest conditions and tested them in a standard environmental condition (16,30).

Some studies demonstrated that the olfactory ability of a human reaches a plateau between the ages of 20 and 60, with subjects aged less than 20 years or more than 60 years of age having lower scores in olfactory evaluations (5,31,32). The effect of neurodegenerative disease or olfactory epithelium damage are common explanations for olfactory impairments in old age (32,33). However, the reasons in children are not obviously defined, and may be the same as the development in their verbal skills (34,35). In our study we selected people aged 20–60 in order to minimize these interfering factors. Although in our study women had lower MICs in more odorants compared with men, these differences were only significant for hazelnut. Surprisingly, men had a lower MIC compared with women for this odorant. However, some authors considered sex hormones to have the ability to affect the olfactory function. In a systematic review by Doty et al. (36), women were shown to have a higher correct identification rate compared with men for some odorants, especially for body odors. However, for more accurate analysis, situations such as pregnancy, use of oral hormonal contraceptives, or menstrual cycle need to be considered (37–39).

The differences in MICs across the age subgroups were also not significant (except for lemon). These data show that these MICs can be used sex- and age-independently in a SIT. 

## Conclusion

In the present study, we tried to introduce some culturally-familiar odorants with their MICs for use in the SIT. However, the detection of an odorant may also be influenced by the different genetic and environmental factors. As Iran consists of various ethnic subgroups, this selection of odorants needs to be evaluated in a cross-country study including subjects from various ethnic subgroups.
